# Effects of Toxic Compounds in *Montipora capitata* on Exogenous and Endogenous Zooxanthellae Performance and Fertilization Success

**DOI:** 10.1371/journal.pone.0118364

**Published:** 2015-02-25

**Authors:** Mary Hagedorn, Ann Farrell, Virginia Carter, Nikolas Zuchowicz, Erika Johnston, Jacqueline Padilla-Gamiño, Sarath Gunasekera, Valerie Paul

**Affiliations:** 1 Department of Reproductive Sciences, Smithsonian Conservation Biology Institute- National Zoological Park, Washington, District of Columbia, United States of America; 2 Hawai’i Institute of Marine Biology, University of Hawaii, Kaneohe, Hawaii, United States of America; 3 Smithsonian Marine Station, Fort Pierce, Florida, United States of America; University of New South Wales, AUSTRALIA

## Abstract

Studies have identified chemicals within the stony coral genus *Montipora* that have significant biological activities. For example, Montiporic acids A and B and other compounds have been isolated from the adult tissue and eggs of *Montipora spp.* and have displayed antimicrobial activity and cytotoxicity in cultured cells. The ecological role of these toxic compounds is currently unclear. This study examines the role these toxins play in reproduction. Toxins were found in the eggs and larvae of the coral *Montipora capitata*. Releasing these toxins by crushing both the eggs and larvae resulted in irreversible inhibition of photosynthesis in endogenous and exogenous zooxanthellae within minutes. Moreover, these toxins were stable, as frozen storage of eggs and larvae did not affect toxicity. Photosynthetic competency of *Porites compressa* zooxanthellae treated with either frozen or fresh, crushed eggs was inhibited similarly (P > 0.05, ANCOVA). Addition of toxic eggs plugs to live *P. compressa* fragments caused complete tissue necrosis under the exposed area on the fragments within 1 week. Small volumes of *M. capitata* crushed eggs added to sperm suspensions reduced *in vitro* fertilization success by killing the sperm. After 30 min, untreated sperm maintained 90 ± 1.9% SEM motility while those treated with crushed eggs were rendered immotile, 4 ± 1.4% SEM. Flow cytometry indicated membrane disruption of the immotile sperm. Fertilization success using untreated sperm was 79 ± 4% SEM, whereas the success rate dropped significantly after exposure to the crushed eggs, 1.3 ± 0% SEM. Unlike the eggs and the larvae, *M. capitata* sperm did not reduce the photosynthetic competency of *P. compressa* zooxanthellae, suggesting the sperm was nontoxic. The identity of the toxins, cellular mechanism of action, advantage of the toxins for *M. capitata* and their role on the reef are still unknown.

## Introduction

Adaptation is a key component to species survival. The field of marine chemical ecology continues to unravel the complex ecological functions of marine natural products. These compounds feature heavily in predator–prey interactions, trophic cascades, competition, prey capture, reproduction, and larval recruitment, and have added to ecological theory [1]. Competition for space is an important ecological process on coral reefs, and Pawlik et al. [2] found that sponge compounds may stress corals by adversely influencing their symbiotic zooxanthellae.

Allelopathy is one such adaptation where natural chemical defenses, such as toxins, inhibit the growth of another cell. Allelopathic compounds may have complex chemical structures and significant biological activities. They have been isolated from almost all phyla of marine organisms [1,3]. The most commonly known producers of toxins in the marine environment are marine algae, soft corals, sponges and gorgonians [1,2,4]. Little is known about allelopathy in stony corals, but some studies have identified a variety of chemicals within the genus *Montipora* that have significant biological activities. For example, Montiporic acids A and B and other compounds have been isolated from the eggs of *Montipora* spp. and have displayed antimicrobial activity and cytotoxicity in cultured cells [5–9]. In addition, Gochfeld and Aeby [10] tested *Montipora capitata* extracts for their antimicrobial activity and found that extracts of this coral inhibited up to 54% of bacterial strains, including known coral pathogens found in the surrounding seawater. *Montipora* [11] is the second largest genus in the family Acroporidae and has a cosmopolitan distribution [12], so if these adaptations are shared by many of the species within the genus, it could have broad implications for survival and adaptation on reefs around the world.

The broad antimicrobial capability within *Montipora* tissue was known, however, in preliminary studies on the physiology of zooxanthellae from *M*. *capitata* [13], we observed something far more deadly. Specifically, the zooxanthellae from the tissue of three Hawaiian coral species were successfully harvested from host tissues and remained viable for days, whereas when *M*. *capitata* zooxanthellae were extracted, photosynthesis ceased within minutes. Moreover, when either fresh, boiled (100°C for 15 min) or frozen adult *M*. *capitata* tissue was added to samples of the viable exogenous zooxanthellae from other species, photosynthesis ceased in minutes. This suggested the presence of toxic compounds within *M*. *capitata* tissue.

**Fig 1:**
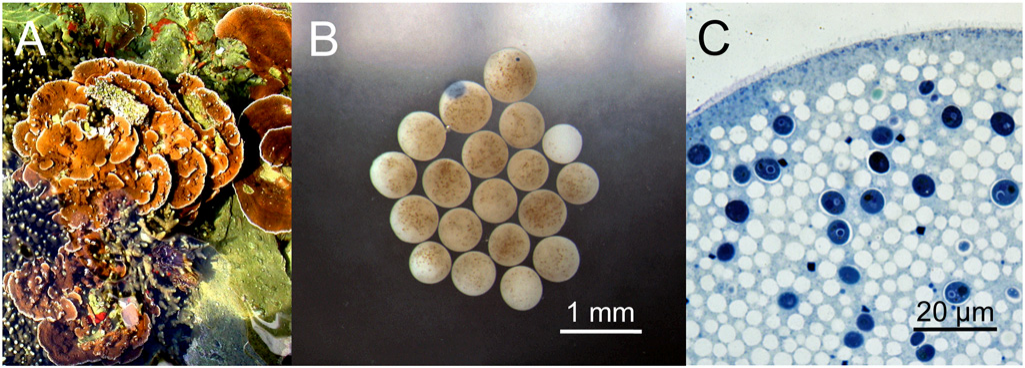
Morphology of *M*. *capitata*. (**A)** Plate-like adult *M*. *capitata* found in Hawaii. **(B)** Light micrograph of M. *capitata* eggs demonstrating size and appearance of vertically transmitted intracellular zooxanthellae. **(C)** Transmission electron micrograph of a single *M*. *capitata* egg demonstrating the size and appearance of white yolk droplets and deep blue membrane-bound endogenous zooxanthellae.

*M*. *capitata* is found in the shallow areas of Kaneohe Bay, Hawaii, and assumes a plate-like form (Fig. 1A). Adult *M*. *capitata* transfer zooxanthellae horizontally into their eggs prior to spawning (Fig. 1B and C). During the massive spawns, *M*. *capitata* egg/sperm bundles form large slicks, which can be easily damaged by wind and waves potentially releasing a toxic cocktail. To examine this further, we investigated the role toxins might play in *M*. *capitata* reproduction over the 3-month spawning period. In this paper, we examined: (1) the toxic affects within the eggs, sperm and larvae on endogenous and exogenous zooxanthellae; (2) the stability of the toxins to freezing; (3) whether the toxins can adversely affect live zooxanthellae in intact *Porites compressa* fragments; (4) the impact the toxic compounds in the eggs might have on sperm motility, viability and *in vitro* fertilization; and, (5) the toxicity of various coral-derived organic chemical fractions against zooxanthellae.

## Materials and Methods

### Coral Collection and Husbandry

*M*. *capitata*, *P*. *compressa* and *Fungia scutaria* colonies were collected from various shallow reef flats around Coconut Island in Kaneohe Bay, Hawaii throughout the summer, and then maintained in shallow running seawater tables at the Hawaii Institute of Marine Biology, University of Hawaii. Care was taken to choose colonies from different locations in the bay, so as to try to ensure as much genetic diversity as possible. Colonies were kept in flowing seawater tables, supplied by water directly drawn from Kaneohe Bay. *M*. *capitata* spawns on the new moon throughout the summer in Hawaii [14–16], and the captive *M*. *capitata* fragments were used mainly for collecting gametes. Collection was performed with the appropriate permits from the state of Hawaii’s Department of Land and Natural Resources (Special Activity Permit # SAP 2011–1). No ethical approval was required for any of the experimental research described herein.

Egg/sperm bundles were collected in June, July and August of 2011 and June and July of 2012 and either used fresh or flash frozen in liquid nitrogen, then stored frozen at-80°C. To rear larvae, two egg/sperm bundles from different individuals were placed into 10 ml of filtered seawater (FSW) in 20 ml glass vials and allowed to develop for 3 days. Each vial produced about 100 larvae, and many vials were combined to produce enough tissue for experiments.

### Microscopy

Samples for transmission electron microscopy were processed according to Padilla-Gamiño et al. [17]. In brief, specimens were fixed with 4% glutaraldehyde in 0.1 M sodium cacodylate buffer for 48 h, washed in 0.1 M sodium cacodylate, followed by post-fixation with 1% OsO_4_ in 0.1 M sodium cacodylate buffer for 1h. Tissue was dehydrated in a graded ethanol series and embedded in LX112 epoxy resin. Ultrathin (60–80 nm) sections were cut with a Reichert Ultracut E ultramicrotome, double stained with uranyl acetate and lead citrate, viewed on a LEO 912 EFTEM at 100 kV, and photographed with a Proscan frame-transfer CCD.

### Monitoring Zooxanthellae

The presence of intracellular zooxanthellae is critical to the health of the coral polyp. Therefore, zooxanthellae number and robustness of photosynthetic performance were monitored over time using a Pulse Amplitude Modulated fluorometer (PAM, Walz, Germany) to determine relative levels of photosynthesis in photosystems I and II between the treatments and controls. Briefly stated, the PAM measured the initial fluorescence (recorded as “F”), then administered a saturating pulse of light, recording the maximal fluorescence (recorded as “M”). A ratio of these, F/M, yielded the effective quantum yield, recorded as the “Y-value”. We reported the effective quantum yield or Y-value throughout. The samples were not dark acclimated, but recorded under fluorescent lighting in the laboratory at mean light level of 4.08 ± 0.29 µmols/m^2^/s SEM (n = 5). Zooxanthellae were harvested from their host as described in Hagedorn et al. [13]. Unless stated otherwise, all solutions were made with 0.2 µm- FSW for these studies. A reduction in the effective quantum yield (Y) correlated with dying zooxanthellae [13]. These experiments mixed dead and live zooxanthellae to produce a known live/dead yield curve [13].

Exogenous zooxanthellae extracted from two different coral species (*F*. *scutaria* and *P*. *compressa*) were used for these experiments. *M*. *capitata* zooxanthellae could not be extracted successfully, so the only physiological examination of *M*. *capitata* zooxanthellae was of the endogenous zooxanthellae vertically transmitted into the eggs and larvae.

### Experiments

**Experiment 1: Assessing the effect of damaged *M*. *capitata* eggs and larvae on endogenous zooxanthellae.** Two equal sub-samples (0.5 ml) of egg-sperm bundles were taken from each *M*. *capitata* colony (n = 7) diluted with FSW to a total volume of 1.0 ml per sample. For each pair, one sub-sample was designated as the control (no treatment) and the other as the experimental (crushed).

The viability of both samples was evaluated with the PAM fluorometer for effective quantum yield at t = 0. Then, the experimental sample was homogenized with ten strokes in a glass homogenizer. The time count was started at the moment that the homogenization of the sample began. Fluorescence was measured in both the control and the experimental samples at t = 1, 2, 3, 4, 5, and 10 min. PAM data were acquired with the fiber optic tip held in the center of the 1 ml sample in a 1.5 ml microcentrifuge tube at 26.5°C.

To determine whether the toxicity remains during development, parallel crushing experiments were done with 4 day-old *M*. *capitata* larvae. Thousands of larvae were reared in 20 ml glass scintillation vials for 4 days from 4 specific crosses. The larvae from identical crosses were consolidated then divided into two sub-samples, diluted in FSW yielding a final volume of 500 µl.

**Experiment 2: Assessing stability of the toxins after frozen storage.** Small fragments (10 x 3 cm; n = 10 individuals) of *P*. *compressa* were collected from around the Hawaii Institute of Marine Biology, placed in a flowing seawater table for a few days, and then their zooxanthellae were extracted following previous methods [13]. The extracted zooxanthellae sample from each colony was split into two sub-samples for parallel exposure to fresh or frozen/thawed egg tissue. One ml samples of *M*. *capitata* eggs with as little seawater as possible were frozen in liquid nitrogen, then thawed in a 30°C water bath; this process was repeated three times. Both the fresh and thawed eggs were crushed in a glass homogenizer, using 10 to 15 strokes. Each *P*. *compressa* subsample consisted of 500 µl of *P*. *compressa* zooxanthellae at ~10^6^ cells/ml to which 75 µl of either fresh or frozen crushed *M*. *capitata* eggs were added. The effect on the quantum yield was monitored over time (t = 5, 10, 15 and 30 min) by PAM.

To establish the toxicity threshold, a dose response curve was determined using frozen/thawed eggs from mixed *M*. *capitata* colonies (n = 10 individuals). Eggs from *M*. *capitata* were crushed (as described above) and 25, 50, 100 or 250 µl of tissue added to 500 µl of *P*. *compressa* zooxanthellae at ~10^6^ cells/ml (n = 1 individual). PAM measurements were taken over 60 min.

To confirm the optimal dose determined above, paired, matched samples (as described above) were collected for 8 different *P*. *compressa* colonies, and 100 µl of crushed eggs was added to one sub-sample while 100 µl of FSW was added to the second sub-sample and their quantum yield was monitored over 30 min with a PAM.

**Experiment 3: Assessing the effect of frozen, crushed *M*. *capitata* eggs, sperm and larvae on exogenous zooxanthellae.** Zooxanthellae were extracted and cleaned from eight ~5 cm individual fragments of *P*. *compressa* (as described above). Zooxanthellae samples from each fragment were diluted to 2 x 10^6^ and divided into five 1 ml sub-samples.

Prior to treatment, all *P*. *compressa* zooxanthellae samples were examined with a Junior-PAM (Walz) to obtain baseline effective quantum yield values (time 0). Samples from each individual were then treated with the following matrices: 1) 100 μl of FSW; 2) 100 μl of crushed *F*. *scutaria* sperm at 3x10^8^; 3) 100 μl of crushed *M*. *capitata* sperm at 3x10^8^; 4) 100 μl of *M*. *capitata* eggs packed as tightly as possible that were mixed 1:1 with filtered seawater and crushed; and, 5) 100 μl of *M*. *capitata* larvae that were also packed as tightly as possible and mixed 1:1 with filtered seawater and crushed. After solutions were added, each sub-sample was monitored using PAM with quantum yield values recorded at t = 1, 2, 5, 10, 20, 30 and 40 min.

**Experiment 4: Assessing the effect of frozen *M*. *capitata* eggs on the viability of live zooxanthellae and tissue in *P*. *compressa* fragments.** To investigate whether the toxins in *M*. *capitata* eggs and larvae might assist in larval settlement by clearing space for growth on the reef, *P*. *compressa* fragments were exposed to small volumes of frozen, crushed *M*. *compressa* eggs, frozen crushed *F*. *scutaria* larvae (negative control) and FSW (negative control). Small *P*. *compressa* fragments (10 cm x 3 cm; n = 15 individuals) were collected around the Hawaii Institute of Marine Biology and held in flowing seawater tables. Specifically, 400 µl of crushed eggs, larvae or FSW were added to the caps of 1.5 ml plastic Eppendorf tubes, flash frozen in the caps, stored at-80°C for 1 to 3 weeks and then thawed at 30°C prior to use. Prior to use, each cap was pierced on two opposite sides with a dremel tool, and a plastic ziptie tightly inserted preventing any solution leakage. One of each type of cap was strapped onto each *P*. *compressa* fragment (n = 15 individuals). Each fragment with its 3 tethered caps was placed back into a flowing seawater table. After 24 h, the caps were removed and viability of the *P*. *compressa* tissue and zooxanthellae under each cap was assessed. Digital images were taken of each of the 15 fragments 24 h after treatment with a Wild M3 dissecting microscope at 10x. The change in the coral tissue was assessed using computer image analysis with NIH Image J software examining the relative color change under the treatment area or cap, as compared to a nearby-untreated area (control). An average intensity of red, green and blue colors was taken for a specific sized area (measured in pixels) under the treatment caps and an equal sized area of untreated adjacent tissue. The difference between the color intensity of treated tissue and untreated tissue was calculated. Additionally the change in the viability of the *P*. *compressa* zooxanthellae was assessed with a PAM. The mean control viability of the zooxanthellae of each fragment was assessed in 5 untreated areas by measuring the mean quantum yield. This control value was compared with those measured in the treated areas under each cap.

**Experiment 5: Assessing the effect of damaged *M*. *capitata* eggs on *in vitro* fertilization.** Two equal samples of egg-sperm bundles were taken from each *M*. *capitata* colony (n = 18) and placed into 1 ml micro-centrifuge tubes. Each sample comprised 10 egg-sperm bundles, plus FSW to a total volume of 500 µl per sample. The egg-sperm bundles were allowed to fall apart without agitation, and the sperm was gently removed from the bottom of the tube and placed into a clean 1 ml micro-centrifuge tube, and divided in two. One sub-sample received no treatment. The other sub-sample was treated with 5 µl crushed eggs (*M*. *capitata* eggs were crushed with as little seawater as possible) at t = 0, then the motility of both samples monitored at t = 15 and 30 min, following the methods of Hagedorn et al. [18].

After 30 min, the sperm viability was assessed with a flow cytometer, viability being defined as those cells with intact cell membranes, following the methods of Hagedorn et al. [18]. To determine whether the sperm was membrane intact, sperm from 4 different colonies were treated in three different ways, either with 5 µl FSW (positive control), killed by 3 cycles of freeze/thawing (negative control), or treated with 5 µl of crushed eggs for 15 or 30 min (experimental treatment). We used a standard propidium iodide (Invitrogen) assay that exposed cells to a fluorescent dye that intercalates with DNA nucleotides and is usually excluded by intact cells (Garner et al., 1994). Stained cells were analyzed by flow cytometry (Accuri C6, Accuri Cytometers, Inc. Ann Arbor, MI USA), and 10,000 events were analyzed per sample. Additionally, cell numbers were counted to ensure that no drop in numbers occurred during the treatments.

To determine the impact of the toxins on fertilization, we tested untreated sperm and egg-extract exposed sperm in fertilization assays. *M*. *capitata* does not self-fertilize [15], therefore after motility assessments, untreated or egg-treated sperm at 5 x 10^6^ cells/ml was added to a single egg/sperm bundle from a different colony (n = 18 individuals) in 5 ml of FSW in a 20 ml glass scintillation vial. Fertilization was assessed as the number of developing larvae successfully achieving the prawn-chip stage 12 h later. The mean number of eggs in a *M*. *capitata* bundle (15 ± 5) was used to evaluate the percent fertilization success [17]. As a positive control for *in vitro* fertilization, two bundles, each from a different colony, were placed into 5 ml of seawater in a 20 ml glass scintillation vial and allowed to gently fall apart without agitation (n = 39). As a negative control, 1 egg-sperm bundle from a single colony was placed into 5 ml of seawater in a 20 ml glass scintillation vial and allowed to gently fall apart without agitation (n = 15).

**Experiment 6: Chemical analysis of *M*. *capitata* eggs.** Over 80 ml of concentrated *M*. *capitata* eggs were collected during four mass-spawning events in 2011. Most of the water was removed from the eggs, which were then frozen in liquid nitrogen, and stored at-80°C, then combined and freeze-dried. The freeze dried material (13.4 g) was extracted with ethyl acetate (EtOAc)-methanol (MeOH) (1:1) followed by MeOH- water (H_2_O) (4:1). The combined organic and aqueous extract (6.950 g) was partitioned between EtOAc and H_2_O. The aqueous portion was subsequently partitioned between *n*-butanol (*n*-BuOH) and H_2_O. Concentration of these extracts furnished 6.09 g of EtOAc-soluble fraction, 0.069 g of *n*-BuOH-soluble fraction and 0.994 g of H_2_O-soluble fraction. These fractions were fully lyophilized until a yellowish resin or oil remained. All fractions were analyzed by proton nuclear magnetic resonance spectroscopy to determine the presence of secondary metabolites and to assess the structures of known compounds. Sufficient dimethyl sulfoxide was added to bring all the fractions to ~ 3% solutions (wt/vol) in FSW. *F*. *scutaria* zooxanthellae (n = 2) were removed from adult tissue, cleaned and resuspended in 1 ml sub-samples in FSW at 10^7^ cells/ml. Then 3, 30 or 300 µl of zooxanthellae solution was removed and replaced with either dimethyl sulfoxide (control for carrier), one of the three fractions, frozen, crushed *M*. *capitata* eggs (negative positive control for activity) or FSW (positive negative control for activity). The quantum yield (Y) was monitored over 40 min, and the data were normalized for comparison.

### Statistics

All analyses in this study were performed using Graphpad Prism 5.0 (San Diego, CA) and Microsoft Excel (version 2007). All correlation analyses, ANOVAs were done with a Tukey’s Multiple Comparison Test or Dunnett’s post-test on various data sets. ANCOVA and linear regression was used to determine the difference between the slopes and Y-intercepts of experimental lines. These tests were identified specifically in the results when reporting the P-value, and those values ≤ 0.05 were considered significant.

## Results

### Experiment 1: Crushing *M*. *capitata* eggs or larvae damaged endogenous zooxanthellae

**Fig 2:**
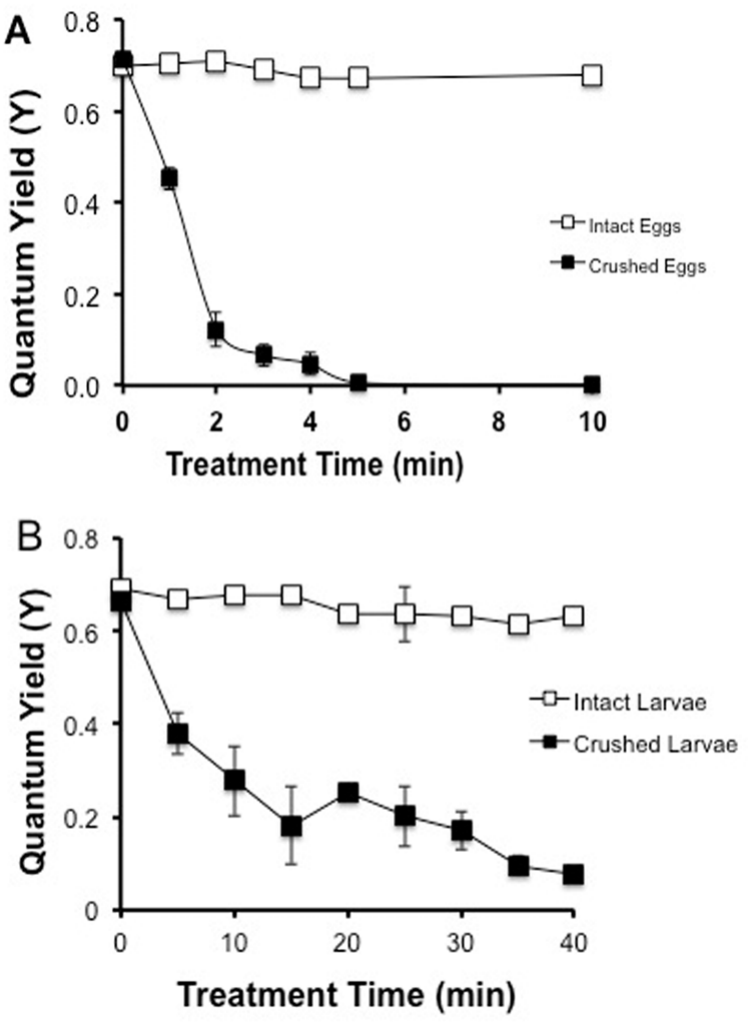
Toxicity of intact and crushed *M*. *capitata* (A) eggs and (B) larvae against endogenous zooxanthellae determined by quantum yield. Bars indicate SEM.

The zooxanthellae of the *M*. *capitata* egg/sperm bundles produced a high quantum yield (Y-value); inferring that they were fully functional. Once the eggs were crushed and their internal zooxanthellae released, the eggs’ own zooxanthellae displayed a Y-value close to zero within 5 min (Fig. 2A). A Y-value close to zero indicated that most of the zooxanthellae in the population were severely inhibited or dead [13]. A similar pattern was observed for *M*. *capitata* larvae, whereby the *M*. *capitata* zooxanthellae displayed a Y-value close to zero within 15 to 30 min (Fig. 2B). Samples (50 to 500 larvae) from 4 different crosses were split into two equal sub-samples and were either entirely crushed or left intact as an untreated control. The untreated group showed no change in its quantum yield over time (P> 0.05, Linear Regression, ANCOVA), whereas the crushed group showed a 90% loss in their quantum yield within 40 min; the two groups have different slopes (P <0.05, ANCOVA, F = 37.7). The time to full inhibition of *M*. *capitata’s* endogenous zooxanthellae was slightly longer for the larvae than the crushed eggs, perhaps due to the difference in the concentration of the eggs versus the larvae in the samples. Even though the times to inhibition were slightly different, it was concluded that damaged eggs and larvae were both capable of inhibiting or damaging endogenous zooxanthellae within minutes, and that the toxins persisted in the larvae prior to settlement.

### Experiment 2: Frozen storage does not affect stability of toxins

**Fig 3:**
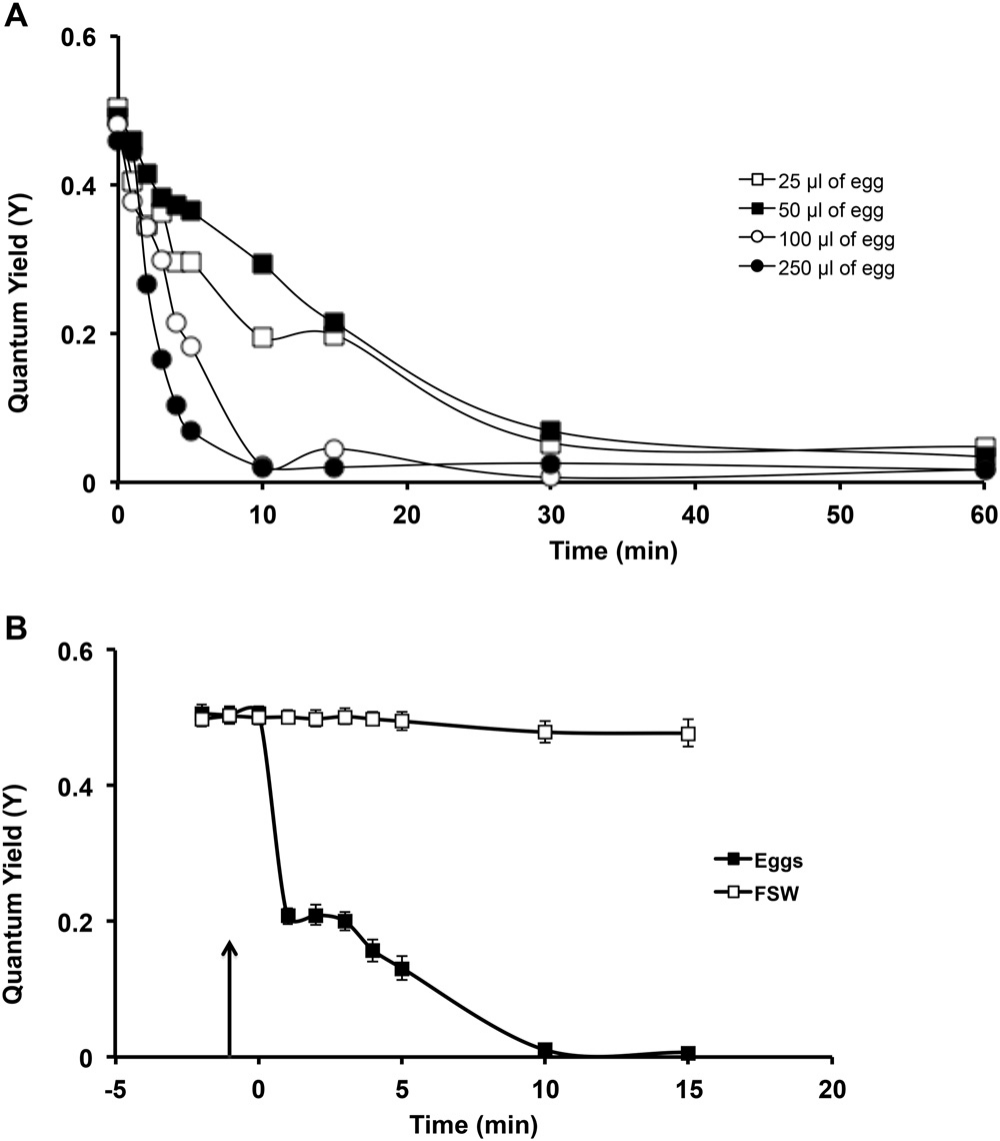
Dose response curve of *M*. *capitata* eggs on exogenous zooxanthellae. **(A)** Preliminary experiments on the dose response curve of the toxins found in *M*. *capitata* eggs were conducted against *P*. *compressa* zooxanthellae. Both 100 and 250 µl of egg volume reduced the *P*. *compressa* zooxanthellae y-values within 10 min, whereas the smaller volumes took 30 min to do the same. **(B)** These experiments were repeated with a larger number of *P*. *compressa* zooxanthellae samples (n = 10 individuals) split into two sub-samples treated with either 100 µl of crushed *M*. *capitata* (at the 0 min mark indicated by the arrow) or 100 µl of FSW. Only the *M*. *capitata* eggs reduced the Y-values. Bars indicate SEM.

A dose response curve of the toxins found in *M*. *capitata* eggs were conducted against *P*. *compressa* zooxanthellae from a single colony. Four previously frozen egg volumes (25, 50, 100 and 250 µl) were added to each volume of fresh *P*. *compressa* zooxanthellae, and the effect on the quantum yield was monitored over 60 min. Both 100 and 250 µl of egg volume reduced the *P*. *compressa* zooxanthellae Y-values within 10 min, whereas the smaller volumes took 30 min to do the same (Fig. 3A). These experiments were repeated with more *P*. *compressa* zooxanthellae samples from10 individuals that were split into two sub-samples and their quantum yield monitored for 2 min, then 100 µl of *M*. *capitata* eggs were crushed and added to one of the subsamples with the other subsample treated with 100 µl of FSW added. After 10 min the zooxanthellae subsamples treated with *M*. *capitata* eggs had a 100% reduction in their Y-values, whereas the FSW-treated were unchanged (Fig 3B).

When parallel sub-samples from the same individuals (n = 10) were tested, both the frozen and the fresh crushed *M*. *capitata* eggs fully inhibited the quantum yield of *P*. *compressa* zooxanthellae within 30 min (data not shown). There was no difference between the slopes (ANCOVA; P > 0.05), demonstrating that the activity of the toxins affected the exogenous zooxanthellae similarly, whether in the fresh or frozen/thawed state.

### Experiment 3: *M*. *capitata* sperm was not toxic to exogenous zooxanthellae

**Fig 4:**
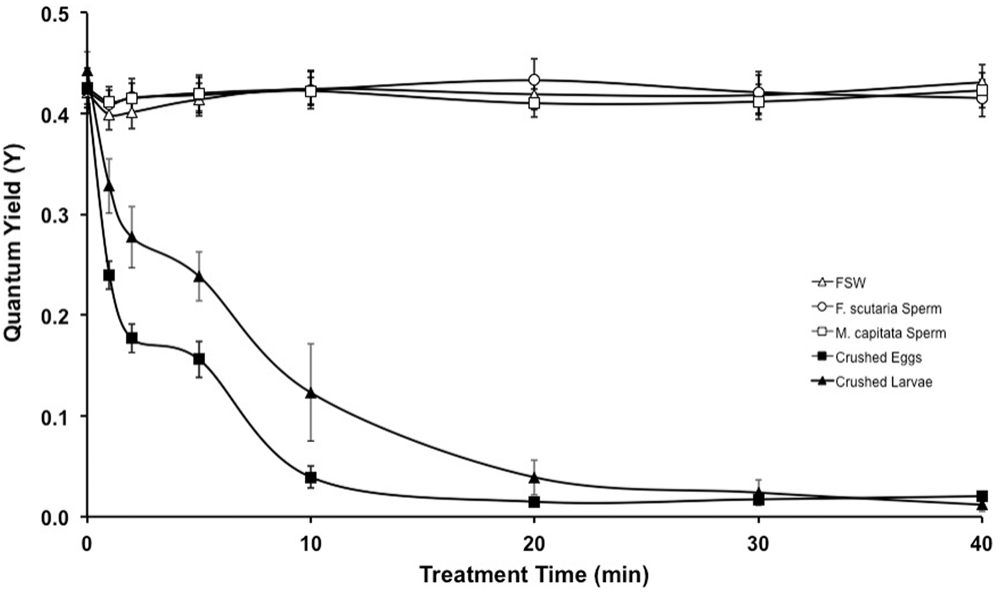
*M*. *capitata* sperm does not contain the same toxic properties as its eggs. *P*. *compressa* zooxanthellae from 8 individuals were split into 5 sub-samples and exposed to five different treatments: 1) frozen/thawed *F*. *scutaria sperm*, 2) frozen/thawed *M*. *capitata* sperm, 3) toxic eggs: frozen/thawed crushed *M*. *capitata* eggs, 4) frozen/thawed crushed *M*. *capitata* larvae, and 5) FSW. After 20 min, the crushed *M*. *capitata* eggs and larvae reduced quantum yield greater than 90% (P < 0.05), while the other treatments remained unchanged at 0.4 (P > 0.05).

*P*. *compressa* zooxanthellae were treated with frozen crushed sperm, eggs, larvae or FSW (Fig. 4). None of these treatments, except the crushed *M*. *capitata* eggs and larvae, affected the quantum yield within the 40 min time period. Specifically, *M*. *capitata* sperm-treated zooxanthellae (n = 8 individuals, P>0.05; Linear Regression, F = 0.004) and the *F*. *scutaria* sperm-treated zooxanthellae (n = 8 individuals, P>0.05; Linear Regression, F = 0.037) were not different than the FSW-treated zooxanthellae (n = 8 individuals, P>0.05; Linear Regression, F = 1.319). In addition, the quantum yield reduced below 0.1 within 20 min for the egg-treated zooxanthellae (n = 8 individuals, P<0.05; Linear Regression, F = 67.93) and the larvae-treated zooxanthellae (n = 4 individuals, P<0.05; Linear Regression, F = 73.01). Although the sperm cells are smaller than the egg cells and not as easily crushed, the repeated freeze/thaw process in combination with the crushing and high cell number should have released some toxins, if present within the cells. These results indicate that the toxins are not located in *M*. *capitata’s* sperm or the concentration is so low that it was not observed in this assay. The reason the *F*. *scutaria* sperm was used in these experiments was to act as a control for cell degradation components from the *M*. *capitata* sperm that might have produced negative effects. Neither sperm addition produced these effects.

### Experiment 4: Crushed *M*. *capitata* eggs adversely affected the tissue and zooxanthellae of live *P*. *compressa*

**Fig 5:**
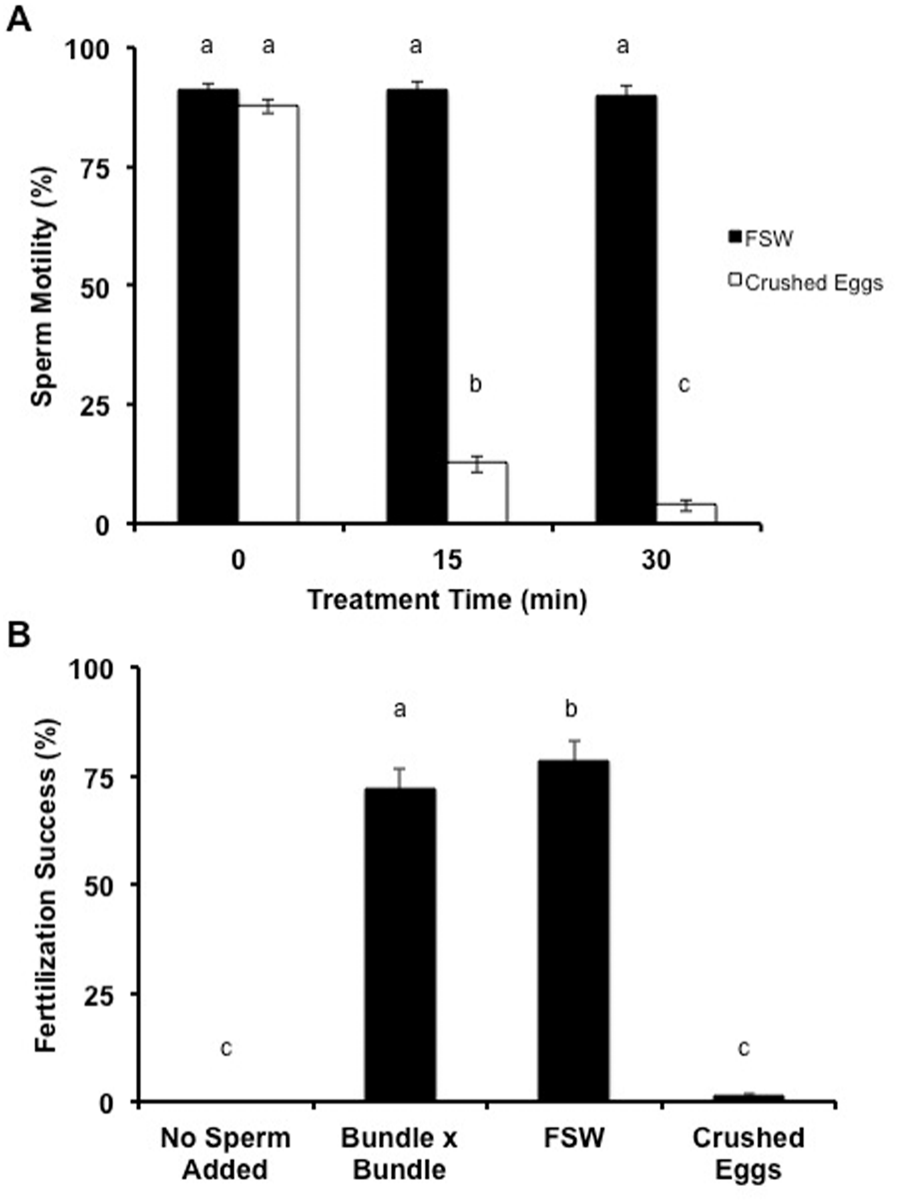
Toxins with the frozen, crushed *M*. *capitata* eggs destroyed the tissue and the zooxanthellae of living *P*. *compressa* fragments. **(A)** Only the addition of the caps with the *M*. *capitata* eggs caused tissue necrosis. **(B, C)** Twenty-four h later, only the caps with the *M*. *capitata* eggs reduced the quantum yield, indicating that the zooxanthellae in those areas were impaired (P< 0.05). Both the *F*. *scutaria* larvae and the *M*. *capitata eggs* caused a changed color on the fragment (P< 0.05), but only addition of the *M*. *capitata* eggs led to complete tissue necrosis under the cap 6 days later. Bars with the same letter indicate (P > 0.05), but bars with different letters indicate (P < 0.05). Bars indicate SEM.

A possible ecological role of the toxins might be to reduce predation; however, this coral is not avoided by coralivores [19]. An alternative hypothesis is that it provides a competitive edge. To test this hypothesis, ‘mock competition’ trials were done by filling Eppendorf caps with either frozen, crushed *F*. *scutaria* larvae, *M*. *capitata* eggs or frozen FSW (Fig. 5A), and attaching one of each of these caps to live *P*. *compressa* fragments (n = 15). Within 24 h the caps with the crushed *M*. *capitata* eggs appeared to damage both the tissue and the zooxanthellae, as measured by a reduction in quantum yield (P< 0.05, F = 386, ANOVA, Tukey’s Multiple Comparison Test, Fig. 5B) and by a color loss under the caps (P< 0.05, F = 44.5, ANOVA, Tukey’s Multiple Comparison Test). This led to tissue necrosis within a week, indicating that the toxins could cause damage to competitors. In contrast, the other treatments caused no long-term damage to the fragments (P< 0.05, F = 45, ANOVA, Tukey’s Multiple Comparison Test, Fig. 5C). The addition of the *F*. *scutaria* larvae did cause a transient color change, but the *P*. *compressa* tissue appeared completely normal 6 days later.

### Experiment 5: *M*. *capitata* eggs adversely affected fertilization success

**Fig 6:**
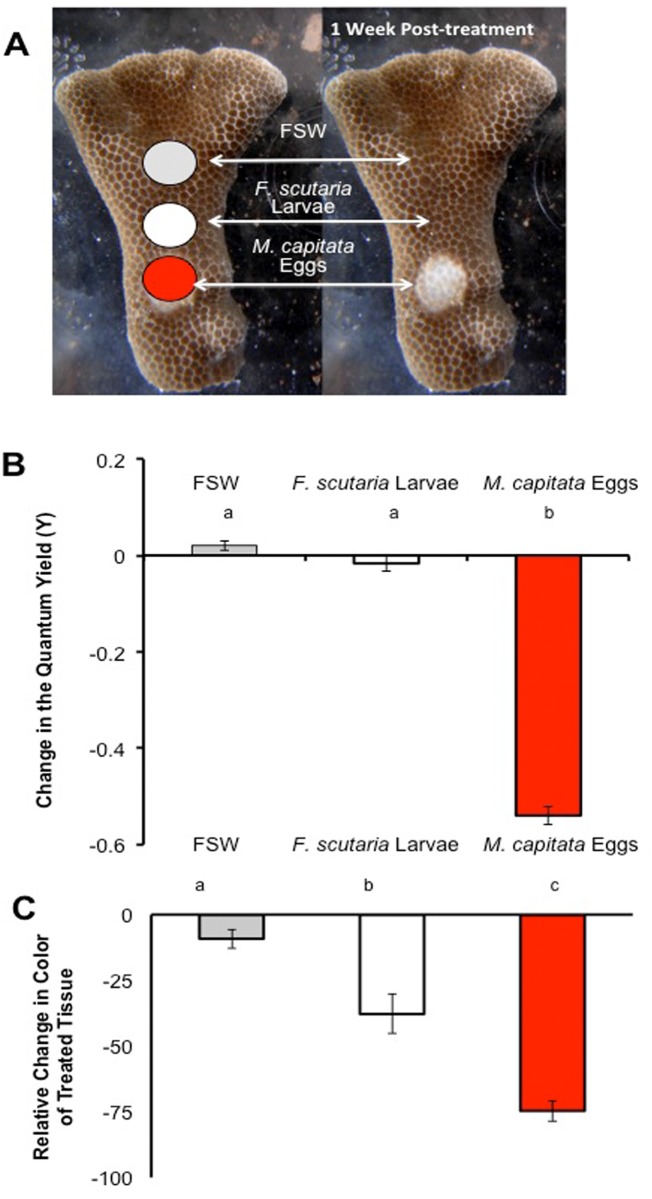
The addition of crushed *M*. *capitata* eggs negatively impacted sperm motility and *in vitro* fertilization success. **(A)** When 5 µl of FSW was added to *M*. *capitata sperm*, its motility remained constant over time, however when 5 µl of crushed *M*. *capitata* eggs were added to *M*. *capitata* sperm, the motility decreased greater than 90% after 15 and 30 min. **(B)** After 30 min, the uncrushed- and crushed egg-treated sperm were tested in *in vitro* fertilization assays. The sperm exposed to the crushed eggs showed little fertilization success (1.3%), whereas the freshly collected bundles from two individuals (positive control) and the uncrushed egg-treated sperm had excellent fertilization success at 72 and 79%. Bars indicate SEM.

Fig. 6A demonstrated that when 5 µl of FSW was added to pair-matched samples of *M*. *capitata* sperm, sperm motility remained constant over time at 90% (n = 12 individuals, P > 0.05, ANOVA, F = 0.2, Tukey’s Multiple Comparison Test); however, when 5 µl of crushed *M*. *capitata* eggs were added to *M*. *capitata sperm*, the motility decreased by more than 90% after 15 and 30 min (n = 12 individuals, P < 0.05, ANOVA, F = 620.9, Tukey’s Multiple Comparison Test). Addition of crushed *M*. *capitata* eggs negatively impacted sperm motility.

To determine whether the sperm was just immotile or whether their membranes were disrupted, crushed egg-treated and FSW-treated sperm were exposed to propidium iodide and examined with a flow cytometer. At 30 min, only 1.2% of the crushed egg-treated sperm cells were membrane intact, whereas 85% of the FSW-treated sperm cells were membrane intact with a mean mortality of only 8.3%. Cell numbers were calculated in each sample. From these data, it was concluded that reduction in viability was not due to an overall loss of cell numbers, but a loss of membrane integrity.

When egg-treated sperm (described above) were used in *in vitro* fertilization assays (Fig. 6B), the sperm exposed to the crushed eggs showed little fertilization success (1.3%), whereas the freshly collected bundles from two individuals (positive control) and the uncrushed egg-treated sperm had excellent fertilization success at 72 and 79% at 30 min, respectively (n = 12 individuals, P < 0.05, ANOVA, F = 4.54, Tukey’s Multiple Comparison Test). Vials with freshly collected bundles from one individual had no fertilization success (negative control).

### Experiment 6: Toxic components are extractable

**Fig 7:**
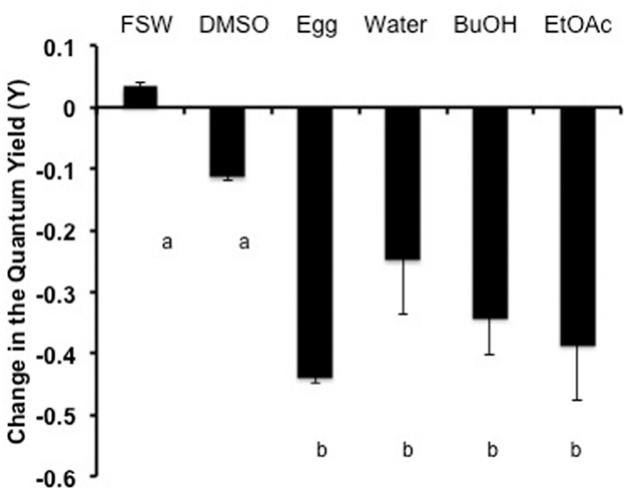
The reduction in the quantum yield of *F*. *scutaria* zooxanthellae due to *M*. *capitata* egg chemical fractions. The zooxanthellae from two individuals were divided up into 5 sub-samples and exposed to 30 µl of FSW, dimethyl sulfoxide (DMSO), frozen crushed *M*. *capitata* eggs, water fraction, butanol fraction (BuOH) or ethyl acetate fraction (EtOAc). The quantum yield of FSW and DMSO, were not different (P>0.05, ANOVA, Dunnett’s Multiple Comparison Test). But adding 30 µl of each of the chemical fractions or frozen eggs caused a reduction of 25 to 44% in the quantum yield over 40 min over the FSW values (P> 0.05, ANOVA, Dunnett’s Multiple Comparison Test). Bars with different letters were (P < 0.05). Bars indicate SEM.

Many known compounds previously reported from *Montipora* spp. could be tentatively identified in the extracts based on ^1^H-NMR. The ethyl acetate extract contained large amounts of the diacetylenes (S1 Fig.), many of which have been previously reported [6,7], and the butanol fraction appeared to contain Montiporic acids A and B, based on the polarity and characteristic signals in the ^1^H-NMR (S2 Fig.) [5]. These compounds have all been reported to have cytotoxic activity. The aqueous extract did not contain these known compounds (S3 Fig.). Methyl montiporate A (S4 Fig.) and montiporyne G (S5 Fig.) were isolated from the complex mixture of diacetylenes in the ethyl acetate fraction. All fractions from the *M*. *capitata* eggs were toxic (Fig. 7), suggesting multiple toxic compounds of different polarities. These showed similar reductions in photosynthetic yields of exogenous zooxanthellae as did the frozen *M*. *capitata* eggs and larvae. Dimethyl sulfoxide was used as a solvent for all the fractions, however at the highest volume tested (300 µl), the solvent alone produced adverse effects on the Y-values of the zooxanthellae (data not shown). While in the 3-µl test volume, neither the solvent nor the coral extracts produced an adverse impact on the zooxanthellae (data not shown). Only the 30-µl test volume produced a reduction in the quantum yield related to the coral extracts (Fig. 7).

## Discussion

Extracts from a number of scleractinian species adversely affect potential pathogens, competitors, and conspecifics, and interestingly, some of these compounds may be seasonally produced [20–22]. For example, extracts of 46 species of adult stony coral from the families of Mussidae, Merulinidae, Siderastreidae, Oculinidae and Dendrophylliidae from the Great Barrier Reef demonstrated bioactivity such as toxicity to mice and fish and antimicrobial activity; however, the antimicrobial activity was only observed prior to reproduction [20]. In contrast, when the adult tissue of six species of soft and stony adult coral were tested in the Red Sea, only soft corals exhibited antimicrobial activity [23]. Additionally, studies have identified antimicrobial activity in the eggs and adult tissue of at least two montiporid species, *M*. *capitata* and *M*. *digitata* [5–10,24]. Both Montiporid species have horizontally transferred zooxanthellae, and one theory is that these toxins may be of plant origin.

Our studies revealed that the toxic components are located in *M*. *capitata* eggs, larvae and adult tissue (data on adults not shown) of *M*. *capitata*. Once the eggs or larvae were crushed, the released toxins were capable of compromising both the zooxanthellae of *M*. *capitata* and those of other species within minutes. But it is not known whether this toxin production is broadly shared throughout the Montiporid complex. The toxins were resistant to freezing and boiling and found in every chemical fraction tested. Coral fragments with zooxanthellae treated with these toxins, produced tissue necrosis within a week’s time. Clearly, the toxins could lead to cell death. Strict parallel experimental exposures on the *P*. *compressa* fragments were not done because we used *F*. *scutaria* larvae and *M*. *capitata* eggs. This was due to timing of the reproduction of the species and availability of material during the fragment experiments. Sperm treated with crushed eggs displayed reduced motility over time and finally membrane disruption at 30 min. This characterization suggests a stable cocktail of chemicals present with *M*. *capitata* adults and offspring capable of producing adverse effects on plant and animal cells.

While the ecological role of the *M*. *capitata* toxins was unclear, the literature points to interspecific competition [19,25]. Toxins may provide a defense against aggressive predation by fish and parasitism by other organisms, but we hypothesize that if the toxins were released, then interspecific competition, and not predator deterrence, may be its major function. Studies on Hawaiian reefs lend support to this hypothesis. They have revealed that coralivorous reef fishes feed freely on *Montipora capitata* with no adverse response [19,25], but when predators were excluded from reef cages, *M*. *capitata* overgrew and outcompeted *P*. *compressa* [19,25]—the dominant species on the reefs in Kaneohe Bay, Hawaii. This edge in competition could be faster growth, or faster growth coupled with chemicals that may inhibit growth in the competing species. Supporting this idea, Gunthorpe and Cameron [21] and Fearon and Cameron [22] found that an aqueous extract from *Goniopora tenuidens* killed the swimming larvae of four different scleractinian corals. Additionally, octocoral allelopathy reduced recruitment and settlement of other corals nearby [26].

Our studies determined that the toxins affected the viability of the sperm and reproductive success of the coral during *in vitro* fertilization, which is commonly done in the investigation of coral reproduction. However, the effects of these toxins may be inferred from previous *in vitro* reproductive studies in *M*. *capitata*. Typically, during *in vitro* fertilization, egg/sperm bundles are collected, and gametes are separated, cleaned, counted and fertilized with typical values of 60 to 80% fertilization success [14]. However, Babcock and Heyward’s method has not worked well for *M*. *capitata* [27]. Instead, only intact bundles from separate individuals placed in small volumes of seawater and allowed to gently fall apart without agitation yielded the typical high fertilization success of 60 to 80% [27]. Even though these toxins inhibited the coral, it is not likely that they might actually impact natural reproduction on the reef. Wind-generated waves can break up the eggs, thus releasing the toxins onto the reef killing sperm and eggs in small areas, but the overall impact to reproductive success over the years would be minimal. Finally, chemical extracts from *M*. *digitata* eggs produced species-specific chemotaxis to sperm. So, the eggs may produce a wide variety of chemicals that have numerous roles in reproduction [28] and defense, potentially leading to greater survival.

An intriguing question is, how does the coral keep these potent toxins from damaging its own tissues and zooxanthellae? We hypothesize that the toxins are stored either in vesicles or diffusely in the cytoplasm, where it may become toxic only after its release. Padilla-Gamiño et al. [17] described a number of types of vesicles within the hydrated eggs of *M*. *capitata* that could potentially sequester these toxins, but more studies would be needed to determine whether, in fact, these toxins are stored there. Other examples of these types of “activated defenses” can be found in the chemical ecology literature for green macroalgae [29,30] and sponges [31].

Reproduction and adaptation are key contributors to species survival. Throughout the world, coral reefs are being degraded at unprecedented rates. Locally, reefs are damaged by pollution, nutrients and sedimentation from outdated land-use, fishing and mining practices [32]. Globally, increased greenhouse gases are warming and acidifying oceans, making corals more susceptible to stress, bleaching and newly emerging diseases [33–35]. The coupling of climate change and anthropogenic stressors has caused a widespread and well-recognized reef crisis [33–38]. However, the types of adaptations that animal populations develop are key to surviving such stressors and depend upon both growth rates and reproduction. The toxic components need to be isolated, identified and then tested to determine which ones elicit which response; only then will their roles become clearer.

## Supporting Information

S1 Fig Ethyl acetate fraction. ^1^H NMR (600 MHz, CDCl_3_) spectrum of ethyl acetate soluble less polar fraction from *Montipora capitata*, indicating the presence of less polar diacetylenes.
(PDF)

S2 Fig *n*-Butanol-soluble fraction. ^1^H NMR (600 MHz, CD_3_OD) spectrum of *n*-Butanol-soluble polar fraction from *Montipora capitata*, indicating the presence of polar diacetylenic montiporic acids.
(PDF)

S3 Fig Water fraction. ^1^H NMR (600 MHz, CD_3_OD) spectrum of water-soluble polar fraction from *Montipora capitata*.
(PDF)

S4 Fig Montiporyne G isolate. ^1^H NMR (600 MHz, CDCl_3_) spectrum of montiporyne G isolated from the ethyl acetate soluble fraction from *Montipora capitata*.
(PDF)

S5 Fig Montiporate A isolate. ^1^H NMR (600 MHz, CDCl_3_) spectrum of methyl montiporate A isolated from the ethyl acetate soluble fraction from *Montipora capitata*.
(PDF)
