# Supplementary material for: Effects of Toxic Compounds in Montipora capitata on Exogenous and Endogenous Zooxanthellae Performance and Fertilization Success
Source: PLoS One. 2015 Feb 25;10(2):e0118364. doi: 10.1371/journal.pone.0118364 (PMC4340954; doi:10.1371/journal.pone.0118364)
Supplement: S1 Fig — 1H NMR (600 MHz, CDCl3) spectrum of ethyl acetate soluble less polar fraction from Montipora capitata, indicating the presence of less polar diacetylenes. (PDF) [file pone.0118364.s001.pdf]

EtOAc–soluble less polar fraction

This fraction indicates the presence of less polar diacetylenes

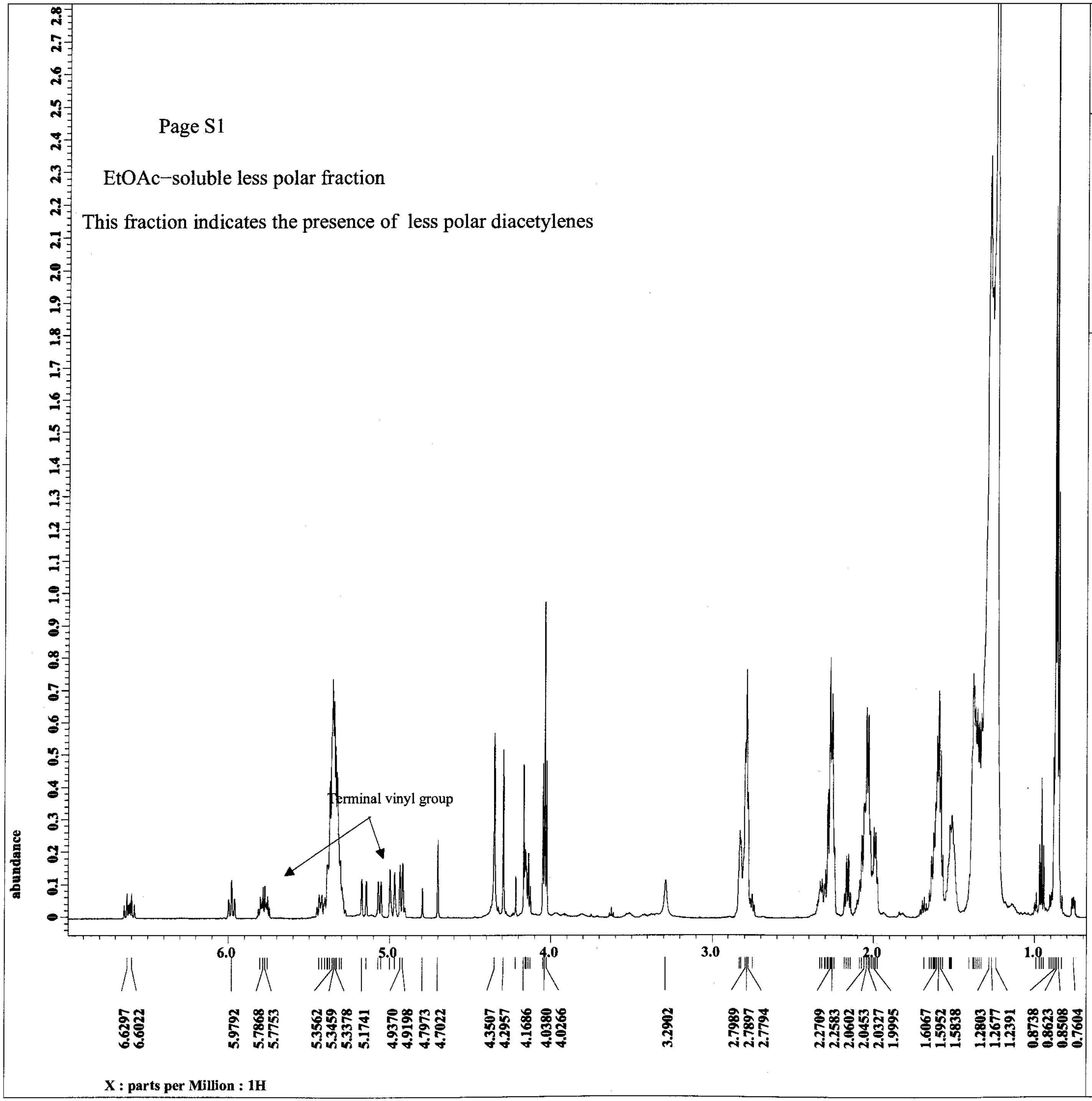


```
---- PROCESSING PARAMETERS ----
dc_balance : 0 : FALSE
sexp : 0.2[Hz] : 0.0[s]
trapezoid3 : 0[%] : 80[%] : 100[%]
zerofill : 1
fft : 1 : TRUE : TRUE
machinephase
ppm

Filename           = 3sg87-1-cdcl3-single_
Author             = Smithsonian
Experiment         = single_pulse.ex2
Sample_id          = 3sg87-1-cdcl3
Solvent            = CHLOROFORM-D
Creation_time      =  2-FEB-2012 10:19:33
Revision_time      = 18-NOV-2014 09:23:58
Current_time       = 18-NOV-2014 09:25:03

Comment            = single_pulse
Data_format        = 1D COMPLEX
Dim_size           = 13107
Dim_title          = 1H
Dim_units          = [ppm]
Dimensions         = X
Site               = ECA 600
Spectrometer       = ECA600-AID

Field_strength     = 14.09636928[T] (600[M
X_acq_duration     = 1.4548992[s]
X_domain           = 1H
X_freq             = 600.1723046[MHz]
X_offset           = 5[ppm]
X_points           = 16384
X_prescans         = 1
X_resolution       = 0.68733284[Hz]
X_sweep            = 11.26126126[kHz]
Irr_domain         = 1H
Irr_freq           = 600.1723046[MHz]
Irr_offset         = 5[ppm]
Tri_domain         = 1H
Tri_freq           = 600.1723046[MHz]
Tri_offset         = 5[ppm]
Clipped            = FALSE
Mod_return         = 1
Scans              = 8
Total_scans        = 8

X_90_width         = 6.6[us]
X_acq_time         = 1.4548992[s]
X_angle            = 45[deg]
X_atn              = 3[dB]
X_pulse            = 3.3[us]
Irr_mode           = Off
Tri_mode           = Off
Dante_presat       = FALSE
Initial_wait       = 1[s]
Recvr_gain         = 36
Relaxation_delay   = 5[s]
Repetition_time    = 6.4548992[s]
Temp_get           = 22.9[dC]
```
