# Supplementary material for: Effects of Toxic Compounds in Montipora capitata on Exogenous and Endogenous Zooxanthellae Performance and Fertilization Success
Source: PLoS One. 2015 Feb 25;10(2):e0118364. doi: 10.1371/journal.pone.0118364 (PMC4340954; doi:10.1371/journal.pone.0118364)
Supplement: S3 Fig — 1H NMR (600 MHz, CD3OD) spectrum of water-soluble polar fraction from Montipora capitata. (PDF) [file pone.0118364.s003.pdf]

Water–soluble polar fraction

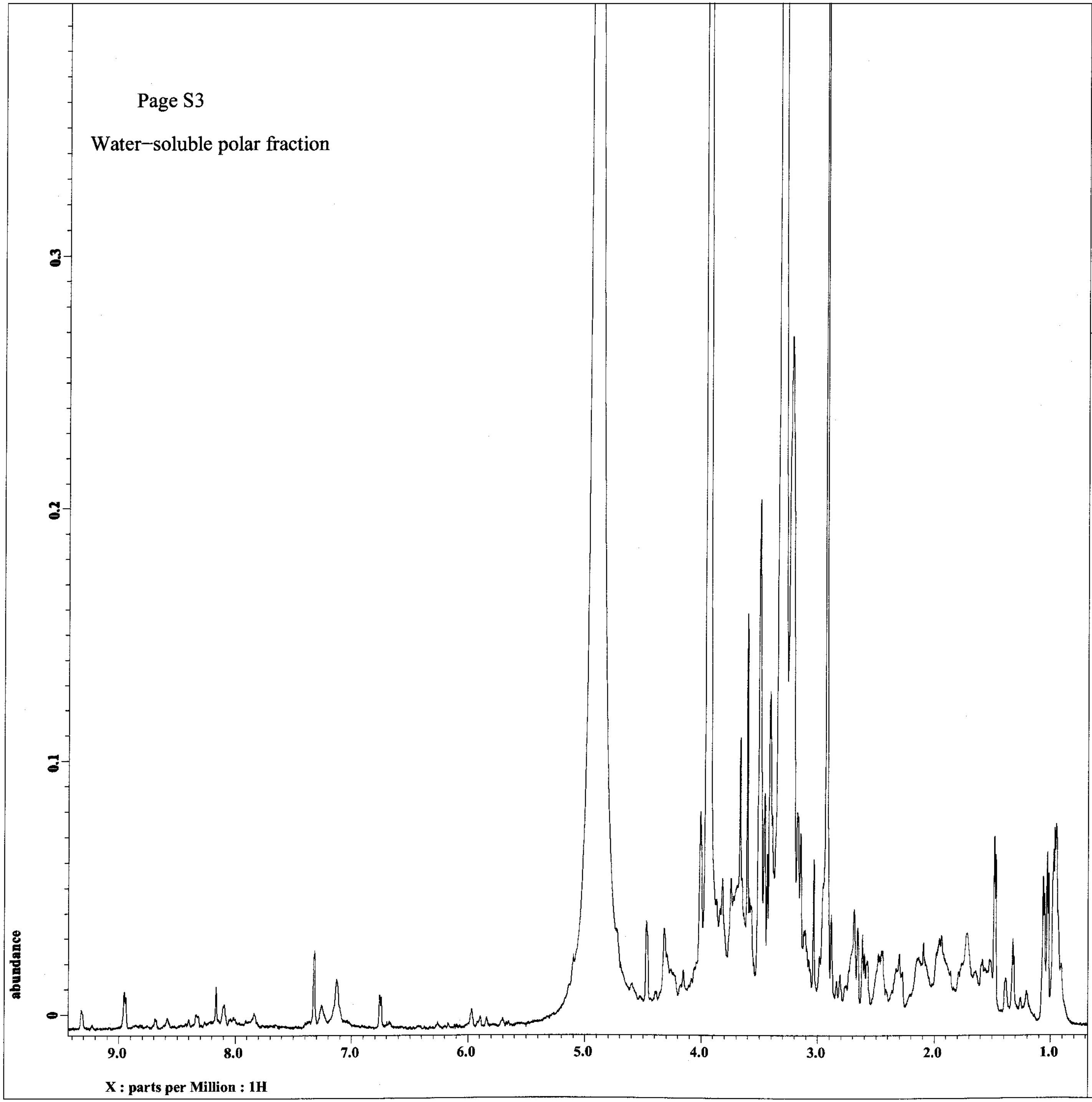


```
---- PROCESSING PARAMETERS ----
dc_balance : 0 : FALSE
sexp : 0.2[Hz] : 0.0[s]
trapezoid3 : 0[%] : 80[%] : 100[%]
zerofill : 1
fft : 1 : TRUE : TRUE
machinephase
ppm
phase : 2 : 0 : 50[%]
```

```
Filename          = 3sg87-3-cd3od-single_
Author            = Smithsonian
Experiment        = single_pulse.ex2
Sample_id         = 3sg87-3-cd3od
Solvent           = METHANOL-D3
Creation_time     =  9-FEB-2012 16:32:13
Revision_time     = 18-NOV-2014 09:35:40
Current_time      = 18-NOV-2014 09:36:22

Comment           = single_pulse
Data_format       = 1D COMPLEX
Dim_size          = 13107
Dim_title         = 1H
Dim_units         = [ppm]
Dimensions        = X
Site              = ECA 600
Spectrometer      = ECA600-AID

Field_strength    = 14.09636928[T] (600[M
X_acq_duration    = 1.4548992[s]
X_domain          = 1H
X_freq            = 600.1723046[MHz]
X_offset          = 5[ppm]
X_points          = 16384
X_prescans        = 1
X_resolution      = 0.68733284[Hz]
X_sweep           = 11.26126126[kHz]
Irr_domain        = 1H
Irr_freq          = 600.1723046[MHz]
Irr_offset        = 5[ppm]
Tri_domain        = 1H
Tri_freq          = 600.1723046[MHz]
Tri_offset        = 5[ppm]
Clipped           = FALSE
Mod_return        = 1
Scans             = 8
Total_scans       = 8

X_90_width        = 6.6[us]
X_acq_time        = 1.4548992[s]
X_angle           = 45[deg]
X_atn             = 3[dB]
X_pulse           = 3.3[us]
Irr_mode          = Off
Tri_mode          = Off
Dante_presat      = FALSE
Initial_wait      = 1[s]
Recvr_gain        = 34
Relaxation_delay  = 5[s]
Repetition_time   = 6.4548992[s]
Temp_get          = 23.1[dC]
```
