# Supplementary material for: Effects of Toxic Compounds in Montipora capitata on Exogenous and Endogenous Zooxanthellae Performance and Fertilization Success
Source: PLoS One. 2015 Feb 25;10(2):e0118364. doi: 10.1371/journal.pone.0118364 (PMC4340954; doi:10.1371/journal.pone.0118364)
Supplement: S4 Fig — 1H NMR (600 MHz, CDCl3) spectrum of montiporyne G isolated from the ethyl acetate soluble fraction from Montipora capitata. (PDF) [file pone.0118364.s004.pdf]

Purified sub–fraction from EtOAc–soluble non polar fraction

Major compound in this fraction

Methyl montiporate A

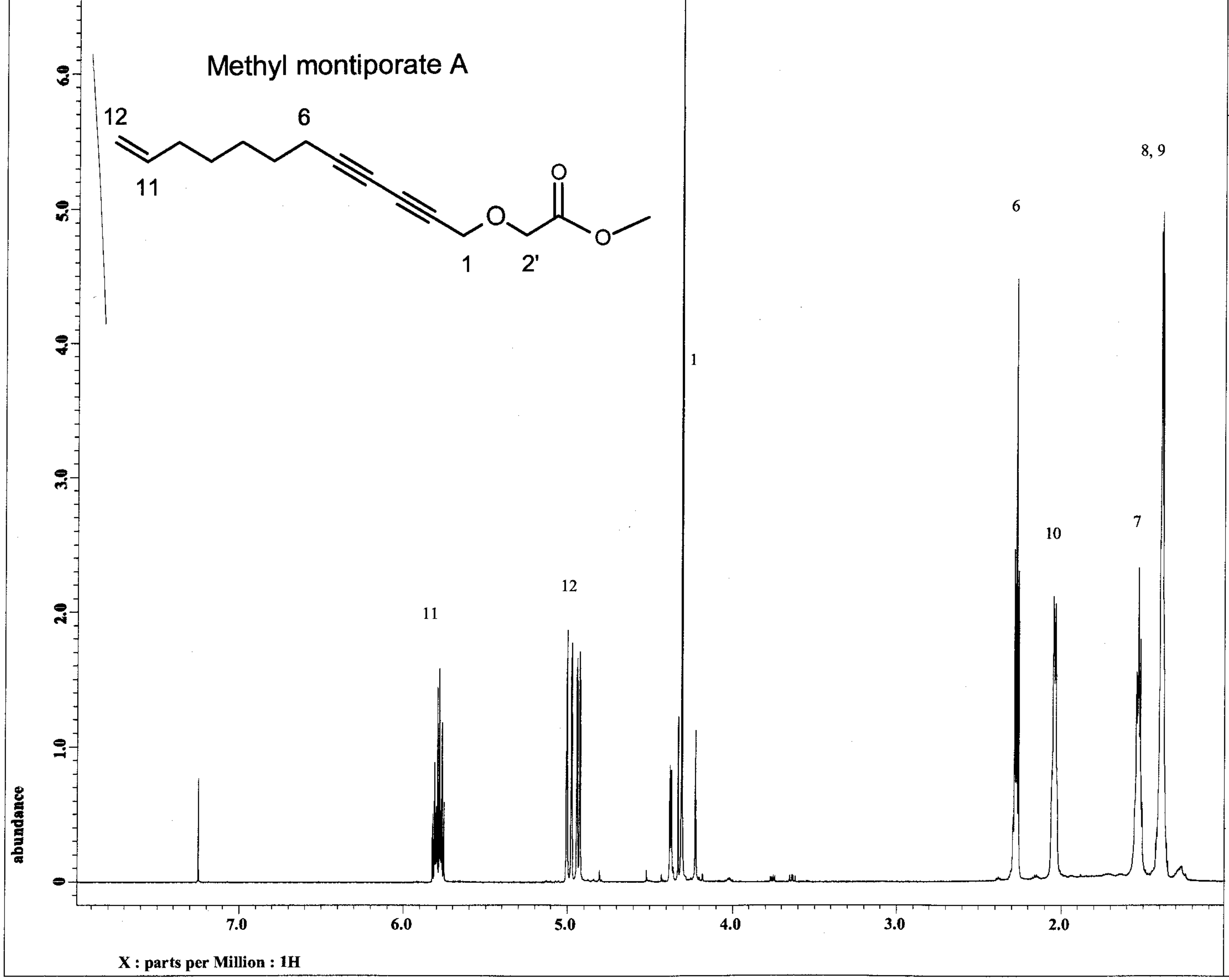


```
---- PROCESSING PARAMETERS ----
dc_balance : 0 : FALSE
sexp : 0.2[Hz] : 0.0[s]
trapezoid3 : 0[%] : 80[%] : 100[%]
zerofill : 1
fft : 1 : TRUE : TRUE
machinephase
ppm

Filename            = 3sg92-2-cdcl3-single_
Author              = Smithsonian
Experiment          = single_pulse.ex2
Sample_id           = 3sg92-2-cdcl3
Solvent             = CHLOROFORM-D
Creation_time       =  8-MAR-2012 15:38:20
Revision_time       = 18-NOV-2014 09:39:10
Current_time        = 18-NOV-2014 09:39:41

Comment             = single_pulse
Data_format         = 1D COMPLEX
Dim_size            = 13107
Dim_title           = 1H
Dim_units           = [ppm]
Dimensions          = X
Site                = ECA 600
Spectrometer        = ECA600-AID

Field_strength      = 14.09636928[T] (600[M
X_acq_duration      = 1.4548992[s]
X_domain            = 1H
X_freq              = 600.1723046[MHz]
X_offset            = 5[ppm]
X_points            = 16384
X_prescans          = 1
X_resolution        = 0.68733284[Hz]
X_sweep             = 11.26126126[kHz]
Irr_domain          = 1H
Irr_freq            = 600.1723046[MHz]
Irr_offset          = 5[ppm]
Tri_domain          = 1H
Tri_freq            = 600.1723046[MHz]
Tri_offset          = 5[ppm]
Clipped             = FALSE
Mod_return          = 1
Scans               = 8
Total_scans         = 8

X_90_width          = 6.6[us]
X_acq_time          = 1.4548992[s]
X_angle             = 45[deg]
X_atn               = 3[dB]
X_pulse             = 3.3[us]
Irr_mode            = Off
Tri_mode            = Off
Dante_presat        = FALSE
Initial_wait        = 1[s]
Recvr_gain          = 54
Relaxation_delay    = 5[s]
Repetition_time     = 6.4548992[s]
Temp_get            = 24.2[dC]
```
