# Supplementary material for: Effects of Toxic Compounds in Montipora capitata on Exogenous and Endogenous Zooxanthellae Performance and Fertilization Success
Source: PLoS One. 2015 Feb 25;10(2):e0118364. doi: 10.1371/journal.pone.0118364 (PMC4340954; doi:10.1371/journal.pone.0118364)
Supplement: S5 Fig — 1H NMR (600 MHz, CDCl3) spectrum of methyl montiporate A isolated from the ethyl acetate soluble fraction from Montipora capitata. (PDF) [file pone.0118364.s005.pdf]

Compound purified from EtOAc–soluble non polar fraction

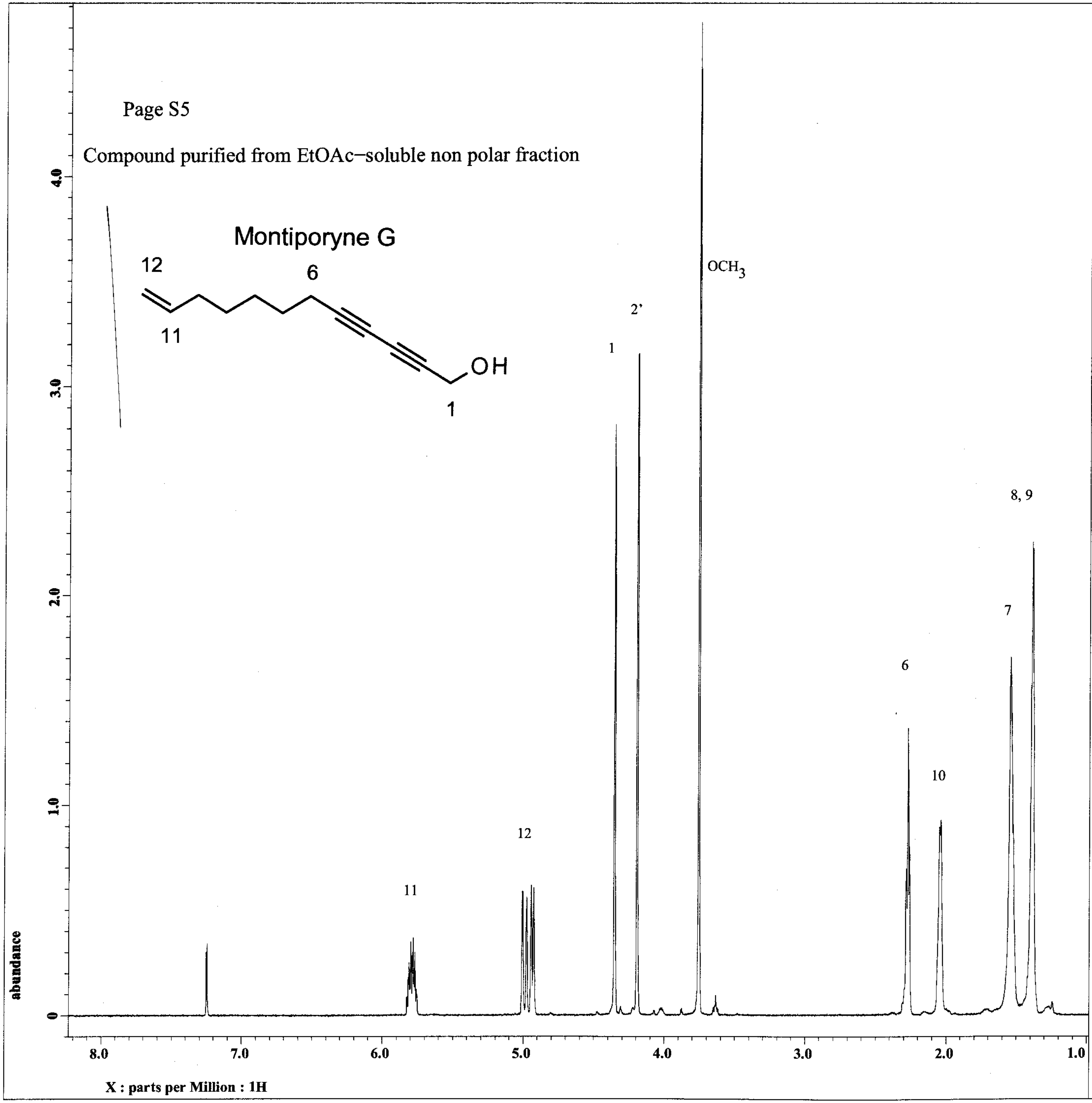

---- PROCESSING PARAMETERS ----
dc_balance : 0 : FALSE
sexp : 0.2[Hz] : 0.0[s]
trapezoid3 : 0[%] : 80[%] : 100[%]
zerofill : 1
fft : 1 : TRUE : TRUE
machinephase
ppm

Filename	= 3sg92-5-cdcl3-single_
Author	= Smithsonian
Experiment	= single_pulse.ex2
Sample_id	= 3sg92-5-cdcl3
Solvent	= CHLOROFORM-D
Creation_time	= 8-MAR-2012 15:55:19
Revision_time	= 18-NOV-2014 09:46:13
Current_time	= 18-NOV-2014 09:46:33
Comment	= single_pulse
Data_format	= 1D COMPLEX
Dim_size	= 13107
Dim_title	= 1H
Dim_units	= [ppm]
Dimensions	= X
Site	= ECA 600
Spectrometer	= ECA600-AID
Field_strength	= 14.09636928[T] (600[M
X_acq_duration	= 1.4548992[s]
X_domain	= 1H
X_freq	= 600.1723046[MHz]
X_offset	= 5[ppm]
X_points	= 16384
X_prescans	= 1
X_resolution	= 0.68733284[Hz]
X_sweep	= 11.26126126[kHz]
Irr_domain	= 1H
Irr_freq	= 600.1723046[MHz]
Irr_offset	= 5[ppm]
Tri_domain	= 1H
Tri_freq	= 600.1723046[MHz]
Tri_offset	= 5[ppm]
Clipped	= FALSE
Mod_return	= 1
Scans	= 8
Total_scans	= 8
X_90_width	= 6.6[us]
X_acq_time	= 1.4548992[s]
X_angle	= 45[deg]
X_atn	= 3[dB]
X_pulse	= 3.3[us]
Irr_mode	= Off
Tri_mode	= Off
Dante_presat	= FALSE
Initial_wait	= 1[s]
Recvr_gain	= 56
Relaxation_delay	= 5[s]
Repetition_time	= 6.4548992[s]
Temp_get	= 24.2[dC]
